# Toward the Personalization of Biceps Fatigue Detection Model for Gym Activity: An Approach to Utilize Wearables’ Data from the Crowd

**DOI:** 10.3390/s22041454

**Published:** 2022-02-14

**Authors:** Mohamed Elshafei, Diego Elias Costa, Emad Shihab

**Affiliations:** Department of Computer Science and Software Engineering, Concordia University, Montreal, QC H3G 1M8, Canada; m_lshafe@encs.concordia.ca (M.E.); diego.costa@concordia.ca (D.E.C.)

**Keywords:** human activity recognition, wearable sensors, wearable sensor data, machine learning

## Abstract

Nowadays, wearables-based Human Activity Recognition (HAR) systems represent a modern, robust, and lightweight solution to monitor athlete performance. However, user data variability is a problem that may hinder the performance of HAR systems, especially the cross-subject HAR models. Such a problem may have a lesser effect on the subject-specific model because it is a tailored model that serves a specific user; hence, data variability is usually low, and performance is often high. However, such a performance comes with a high cost in data collection and processing per user. Therefore, in this work, we present a personalized model that achieves higher performance than the cross-subject model while maintaining a lower data cost than the subject-specific model. Our personalization approach sources data from the crowd based on similarity scores computed between the test subject and the individuals in the crowd. Our dataset consists of 3750 concentration curl repetitions from 25 volunteers with ages and BMI ranging between 20–46 and 24–46, respectively. We compute 11 hand-crafted features and train 2 personalized AdaBoost models, Decision Tree (AdaBoost-DT) and Artificial Neural Networks (AdaBoost-ANN), using data from whom the test subject shares similar physical and single traits. Our findings show that the AdaBoost-DT model outperforms the cross-subject-DT model by 5.89%, while the AdaBoost-ANN model outperforms the cross-subject-ANN model by 3.38%. On the other hand, at 50.0% less of the test subject’s data consumption, our AdaBoost-DT model outperforms the subject-specific-DT model by 16%, while the AdaBoost-ANN model outperforms the subject-specific-ANN model by 10.33%. Yet, the subject-specific models achieve the best performances at 100% of the test subjects’ data consumption.

## 1. Introduction

Fatigue is a natural phenomenon that describes physiological impairments or lack of energy caused by prolonged activities [1]. Lately, a plethora of works in the literature about athlete training and monitoring approaches aim to track body performance and measure muscle fatigue for two reasons: (1) fatigue is an important stamina indicator as previous studies show that it often occurs prior to muscle injuries in over-training [2,3,4] and (2) fatigue-induced injuries are one of the most active research topics in sports science due to their impact on athletes, such as substantial loss in muscle strength and flexibility [5].

Often reported as one of the earliest, the invasive approach is used to detect fatigue by measuring the lactic acid in the bloodstream to determine the maximal muscle effort that a person can maintain without risk of injuries [6,7]. Although such an approach requires puncturing the skin, it often provides accurate information about fatigue conditions and acetate levels in muscles. A previous study requires several blood samples from athletes for a blood lactate test to measure their muscle endurance that can provide accurate results up to 97% [8]. Another previous study that requires blood samples from marathon runners for creatine kinase (CK) tests shows that creatine kinase levels are significantly different (*p*-value < 0.05) in muscles during running, which indicates the risk of skeletal muscle injuries [9]. Less painful but respiratory related, the cardio-respiratory approach monitors a person’s fatigue levels by measuring their circulatory and respiratory systems’ ability to supply oxygen to skeletal muscles during sustained physical exercise without risk of injuries [10,11]. Such an approach may require up to five pieces of equipment such as blood pressure cuff, Electrocardiograph (ECG), bicycle, mouthpiece, and saturation monitor. This setup is complex and requires technical expertise to run; hence, it is considered a drawback for daily living applications. However, it accurately estimates the maximum volume of oxygen consumption $VO_{2}$ during incremental exercises to detect fatigue. A previous study on the oxygen consumption in skeletal muscles measures the respiratory system’s efficiency during sustained physical activity shows that oxygen consumption can provide an accurate indication (>94%) of fatigue intensity [12]. Although the previous approaches may detect signs of fatigue precisely, they often require medical equipment and field training, which makes them too complicated for ordinary users. Recently viewed as the latest, the wearable approach is used to detect fatigue by utilizing sensory data and machine learning algorithms to estimate rates of perceived exertion (RPE) during physical exercises. After the rapid development of low-power wearables, such an approach often represents a modern way to monitor athlete performance and to prevent fatigue-induced injuries during training [13,14]. Previous work on marathon runners that uses data from the inertial measurement unit (IMU) to classify runners as being not-fatigued or fatigued shows that ROC Curves for the General Runners Models, trained using only the statistical features, range between (0.67 and 0.71) [15].

HAR applications in motion tracking and athletic training have become more prominent as wearable technology and machine learning techniques advance [16,17]. Yet, a common obstacle in such applications is having sufficient data to train the HAR models reliably [18,19]. Training HAR models using insufficient data limits their performance and may even make them impractical for their user base. One way to work around this obstacle is to collect data from a large pool of users and train a cross-subject HAR model. However, this does not guarantee an accurate performance from the cross-subject HAR model because even with sufficient data from a large pool of users, individuals may perform the same activity differently. This increases the inter-subject data variability, which hinders the performance of HAR applications [19]. The inter-subject data variability is often high in places where there is a diverse crowd of users with different physical traits [19,20]. A way to reduce the inter-subject data variability is to address each user separately by collecting data from the user of the HAR application to train a subject-specific HAR model. However, the cost of training a subject-specific HAR model is often prohibitive and requires labeled data from the user [21,22,23]. Therefore, there is an inherent trade-off between cross-subject models (cheaper but less accurate) versus subject-specific models (more expensive and more accurate). Furthermore, such a trade-off often exacerbates in specialized cases in HAR (e.g., muscle fatigue detection), where manual or semi-supervised labeling is usually required [24,25]. As a result, this increases the data cost in the case of the subject-specific models; or, if we want to spare that data cost, we will choose the less accurate option, the cross-subject models. In this work, we attempt to bridge the trade-off between cross-subject and subject-specific models. We propose a personalization approach that improves the performance of the cross-subject model while utilizing a small sample of the user’s data.

To further explain our approach, recent studies show that the personalization of HAR cross-subject models can improve the models’ performance while consuming a part of the test subject’s data less than the subject-specific models [26,27]. The rationale behind the personalization is to prioritize the crowd’s data from whom the test subject is similar. Therefore, we study the physical traits of the crowd to improve personalization of the cross-subject model by adding weights to the training data entries based on the inter-subject similarities between the test subject and the subjects from the crowd [28,29,30,31,32]. We believe that combining similarities measure and personalization can form a personalized model which has a better performance than the cross-subject model and consumes the test subject’s data less than the subject-specific models. Moreover, we further enhance the models’ performance by combining hand-crafted features with some of the suggested ones from the previous studies [15,33,34]. The hand-crafted features are often specific toward the activities of interest, which the models have to detect. This further improves the weighting of the training data based on the similarities between training subjects, the crowd, and the test subject.

To evaluate our approach, we perform a case study on detecting biceps muscle fatigue during gym exercise. The study investigates the increasing inter-subject data variability in data patterns for the same exercise, e.g., bicep curls, among gym-goers due to their physical properties and movements. Sometimes, introducing a new member to the gym may increase the inter-subject data variability between the gym-goers if such a member performs exercises differently from the crowd, wherein this often hinders the HAR models’ performance [35]. A previous study shows that using a subject-specific model to address each subject may overcome the inter-subject variability challenge, therefore improving the model’s performance [36]. However, these models are known for their high demand for the subject’s data which is its major drawback [18]. Moreover, these models often suffer from dramatic performance drops in real-time applications if the subject’s data are unavailable or insufficient [37,38].

Detecting bicep muscle fatigue is important because bicep muscle is one of the most active skeletal muscles in the elbow joint [39,40]. Moreover, bicep fatigue injuries, such as muscle strain, may require up to 22 weeks of treatment [41,42], while tendon rupture results in a substantial permanent decrease in flexion and extension strength [43]. This may delay athletes’ training schedules or their immediate withdrawal from competitions [44]. Therefore, given the detrimental effects of bicep muscle fatigue and the low performance of cross-subject detection models, we believe that further methods are needed to improve the performance of the detection models. In our previous work, fatigue detection models achieved accuracies of 98% and 88% for subject-specific and cross-subject models, respectively. However, we observed that as the inter-subject data variability increases, the gap between the performance of cross-subject and subject-specific models widens from 10% to 15% [45]. Therefore, in this work, we propose the personalization of cross-subject models as a solution that provides better performance than cross-subject models while requiring less data than subject-specific models. Moreover, we provide a set of eleven hand-crafted features that enhance the performance of personalized fatigue detection models.

**Figure 1:**
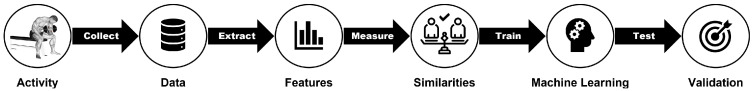
Overview of the similarity-based personalization approach for HAR.

In this work, we employ the personalization of the HAR models to improve the performance of the bicep fatigue detection models. In Figure 1, we show an overview of the approach used in this work. First, we select biceps concentration curls as the activity of interest for this work. Second, we start collecting our data from 25 participants by placing a 50 Hz Neblina inertial measurement unit (IMU) on their wrist. Meanwhile, on the other wrist, we place an Apple Watch Series 4 to measure their heart rate during the exercise. We choose their wrist because it is a primary measuring point for upper limb-related activities. Third, we manually label each repetition in our dataset as containing fatigue or not containing fatigue; then, we compute eleven hand-crafted features commonly reported in the HAR and fatigue-related works. Fourth, we measure two types of similarities, physical and signal similarities between the test subject and the crowd, and we use these similarities to add more weight to data collected from individuals in the crowd who are similar to the test subject. Fifth, we use the weighted dataset to train two machine learning models: Decision Trees (DT) and Artificial Neural Networks (ANN). Finally, we test these models using the test subject’s data in detecting fatigue in biceps concentration curls.

Our first finding shows that it is preferable for the personalized fatigue detection models to depend on both physical and signal similarities with a tendency toward signal similarity. This finding is our first contribution where the eleven hand-crafted features aid the personalization approach in identifying individuals with similar biceps movement patterns to contribute more in the training dataset and hence improving the performance of fatigue detection models. Our second finding shows that the personalization approach improves the accuracy of the DT model by 5.89% and 3.38% for the ANN model. This finding is our second contribution where such improvements occur in personalized cross-subject models after prioritizing training data from the crowd based on the total similarity score, where individuals with high scores contribute more to the models’ training dataset. Our third finding shows that we can further improve the accuracy of personalized models by adding a portion of the test subject’s data to the models’ training datasets. In this case, the personalized DT model achieves 16.0% higher accuracy than the cross-subject DT model while consuming 50.0% less of the test subject’s data than the subject-specific DT model. Moreover, the personalized ANN model achieves 10.33% higher accuracy than the cross-subject ANN model while consuming 33.33% less of the test subject’s data than the subject-specific ANN model. This finding is our third contribution where the personalization approach benefits from the additional data in the training set; yet, it maintains a comparatively lower test subject data consumption by 33.3% up to 50.0% compared to the subject-specific models.

The rest of the paper is structured as follows. Section 2 describes how we collect our dataset and label its entries, as well as illustrates our approach to feature extraction and measure similarities between the test subject and others from the crowd. In Section 3, we present our three experiments and research questions; then, we show the results. In Section 4, we discuss our findings. Section 5 concludes our paper.

## 2. Materials and Methods

This section describes the details of our dataset and its characteristics to represent a sample from the crowd. In addition, we demonstrate the processing steps applied to our dataset to provide a high-quality dataset for our study. Moreover, we extract the eleven features to detect biceps muscle fatigue. Finally, we describe our approach to measure the similarity between a test subject and the crowd.

### 2.1. Dataset Description

**Figure 2:**
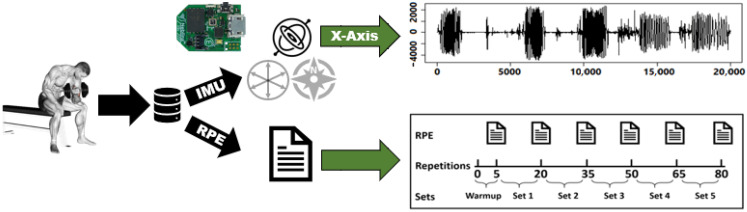
Visualization of data acquisition sessions of biceps concentration curl exercise. Rating of perceived exertion (RPE).

We ask twenty-five volunteers to perform concentration curls while we use the following tools to construct our dataset: (1) We attach a 50 Hz Neblina inertial measurement unit (IMU) to the volunteer’s wrist to measure its acceleration and calculate the linear and angular velocities. Previous studies show velocity loss as an early indicator of muscle fatigue during resistance training, especially when blood lactate and ammonia accumulate in muscle tissues [46,47,48]. (2) We attach an Apple Watch Series 4 to the volunteer’s opposite wrist to measure their heart rate during the exercise. (3) We provide the volunteers with a 4.5 kg weight dumbbell to perform concentration curls. (4) We provide the volunteers with Borg’s scale presented in Table 1 to express their fatigue levels. Such a scale is often used as a subjective method to estimate the rate of perceived exertion (RPE), which expresses the fatigue intensity during an exercise. During data collection sessions, we ask each volunteer to complete 5 warm-up repetitions followed by 15 repetitions per set for a total of 5 sets per hand, as shown in Figure 2. Moreover, the volunteers report their RPE after each set, including the warm-up, yielding 6 RPE values per hand.

It is essential to explain the rationale behind the tools used in the data collection sessions, such as the Apple Watch, the 4.5 kg dumbbell, and the Borg’s scale. Borg’s scale ranges from 6 to 20, where by multiplying these values by ten, we can estimate the volunteer’s heart rate during the exercise. For example, if a volunteer reports 13 on Borg’s scale, we should expect to measure their heart rate around 130 to 140 by the Apple Watch. This serves as a way to strengthen the validity of the reported RPE by each volunteer. However, in the rare cases of dissimilarity between the Borg scale and the measured heart rates, we average the reported RPE with the measured heart rate converted to RPE, as similarly performed in previous work [50]. We use the 4.5 kg weight dumbbell in our work because of three reasons. The first reason is that several previous works have used medium-weight dumbbells ranging between 3.5 kg and 5.5 kg to study muscular strength and fatigue [51,52,53]. The second reason is that medium-weight dumbbells are often reported as the most commonly used dumbbells across gym-goers [54]. Third, in previous work, we found that the 4.5 kg weight dumbbell provides the best trade-off between number data points recorded in data sessions and time to reach fatigue during an exercise [55]. We use Borg’s scale in our work because we believe that RPE is an appropriate marker of fatigue as previous studies within sport science have proven that RPE is capable of modeling a person’s performance better in the real world compared to only heart rate monitoring [7,49,56].

To aptly evaluate our personalization approach, our dataset should contain sufficient data from a diverse set of users performing biceps concentration curls for four reasons. First, with enough data points, we can better capture bicep muscle fatigue, which helps us spot the variations of fatigue patterns among the volunteers. Second, a diverse dataset strengthens our work and better represents a sample from the crowd. The twenty-five volunteers are diverse in age, ranging between 20 and 46, as previous studies show that the selected age covers three distinct stages of athletes’ performance: early, middle, and late, where athletes usually notice physical declines [57,58]. Moreover, the twenty-five volunteers are diverse in weight and height, ranging between 69–127 and 165–190, respectively. It is important to have such variety, as physical characteristics such as weight and height affect arm movements and the severity of injuries, including fatigue-induced ones [59]. We calculated the Body Mass Index (BMI) for the twenty-five volunteers using the following formula [60]:

$$\mathrm{BMI}=\frac{\mathrm{Weight}\left(\mathrm{kg}\right)}{\mathrm{Height}{\left(m\right)}^{2}}$$

**Figure 3:**
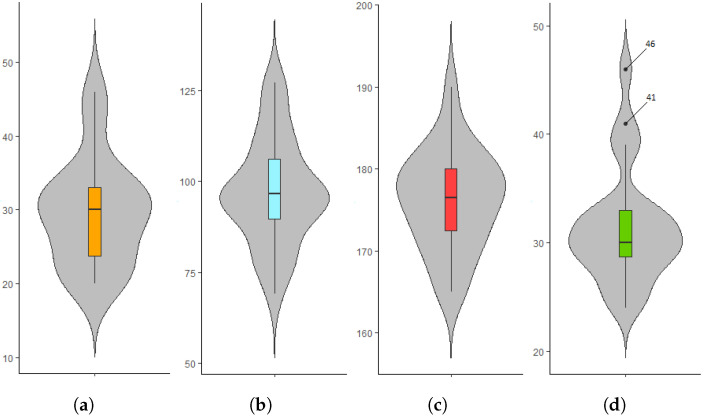
Boxplots to display the distribution of volunteers’ age, weight, height, and BMI in our dataset. (**a**) Age (years). (**b**) Weight (kg). (**c**) Height (cm). (**d**) BMI (kg/m${}^{2}$).

The twenty-five volunteers are also diverse in BMI, ranging between 24 and 46, to include normal weight (18.5≤BMI≤24.9), overweight (25≤BMI≤29.9), and obesity (30<BMI) [61]. The two black dots in Figure 3d are for two volunteers with outlier BMI values of 41 kg/m${}^{2}$ and 46 kg/m${}^{2}$ who are considered extremely obese [62]. Previous studies show that the relationship of BMI to injury risk is bimodal, where trainees with the lowest BMIs exhibit the highest injury risks for both genders and across all fitness levels [63,64]. In addition, the volunteers have no chronic diseases, no muscle or bone surgeries, and have been gym-goers for at least 1 year. Moreover, the volunteers are not on prescribed drugs or substances expected to affect their physical performance.

### 2.2. Dataset Processing

**Figure 4:**
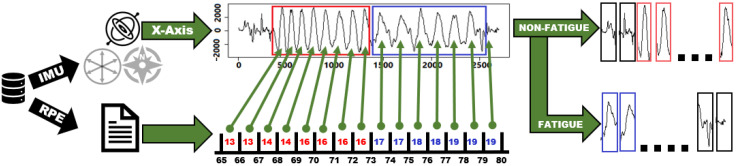
An example of extracting and labeling repetitions of the fifth set from the gyroscope’s x-axis.

Initially, our dataset contains six signals from the two 3D-accelerometer’s signals (*x*, *y*, *z*) and the gyroscope. We start by extracting and labeling the repetitions into fatigue and non-fatigue repetitions. Figure 4 shows an example of the fifth set of repetitions in its raw data form. In this example, the raw data are extracted from the gyroscope’s x-axis along with their corresponding RPE values reported by the volunteer. A previous study on quantifying muscle fatigue suggests an RPE value of 16 as the threshold of true fatigue to estimate the declines in muscle strength during tasks [65]. Therefore, we extract and label each repetition manually according to the RPE values reported for the set, where we label repetitions with reported RPE values larger than 16 as fatigue and others as non-fatigue repetitions. The figure shows two distinct groups of non-fatigue and fatigue repetitions extracted from the set. The troughs indicate full extension, while peaks indicate full flexion. We repeat the same process for the remaining 4 sets.

However, the process is not as exhausting as it may seem per volunteer because we use a synchronized IMU. Therefore, we only need to fully extract and label all 5 sets recorded by one axis per volunteer, e.g., gyroscope’s x-axis. Then, we use the same timestamps from the gyroscope’s x-axis to extract and label repetitions for all the remaining signals (other axes of gyroscope and accelerometer). After we processed all data from the volunteers, our dataset consisted of 3750 repetitions recorded from 6 time-series signals. We compute three additional signals, which are total acceleration, exerted force, and acc–gyro data fusion, as the following:

- Total acceleration: This is the vector sum of the tangential and centripetal accelerations, which makes it a place-independent signal. Hence, total acceleration does not rely on the exact attachment of the accelerometer because it combines *x*, *y*, and *z* acceleration signals at time $t_{i}$ to compute a total acceleration, defined as: $\sqrt{a_{x_{i}}^{2}+a_{y_{i}}^{2}+a_{z_{i}}^{2}}$.
- Exerted force: $F_{exerted}=m\times a$ is the exerted force by the volunteer to lift the dumbbell. $F_{exerted}$ is calculated by multiplying the mass *m* of the lifted dumbbell by acceleration *a*.
- Acc–gyro data fusion (complementary filter): A complementary filter is often used to detect human body movement patterns by combining the gyroscope and the accelerometer [66,67]. Gyroscope’s data are used for precision because it is not vulnerable to external forces, while the accelerometer’s data are used for long-term tracking as it does not drift. We use the Kalman filter algorithm to estimate roll, pitch, and yaw angles [68]. However, we use the yaw angle because it indicates the sideways vibration for the volunteer’s hand during the extension and flexion of the bicep. Previous studies show fatigue may cause a temporary movement disorder, such as skeletal muscles vibration, which indicates fatigue backlogs and increases the vibration angle [69,70,71,72]. In the filter’s simplest form, the equation is defined as: angle=0.98×(angle+gyro×dt)+0.02×acc.

### 2.3. Feature Extraction

Feature extraction is a crucial component of HAR systems because it establishes the most significant parameters to identify or predict human body movements. In addition, feature extraction reduces the data dimensionality while preserving the relevant characteristics of the signal. In this work, we compute a total of eleven hand-crafted features, as shown in Table 2. Eight of the selected features are proven accurate in previous works, especially in the general classification of human activities [15,33,34,73,74,75]. These features include min, max, mean, median, SD, variance, kurtosis, and RMS. Besides the eight features mentioned before, we also select three other features often associated with fatigue for better performance: skewness, IoP, and MSP [76,77,78,79]. A previous study suggests considering the skewness of the data when detecting fatigue in repetitive muscle movements such as bicep curls [55]. We select skewness as a fatigue feature because, during the repetitions’ extraction and labeling process, we observed the following: (1) Non-fatigue repetitions are relatively symmetrical during the repetitions’ extraction and labeling process. (2) In contrast, fatigue repetitions are often positively skewed. Another work shows that fatigue often occurs in later sets, which increases the time to complete repetitions of bicep curls while decreasing the force exerted by the muscles [45]. This is observable through the increments of intervals between peaks, e.g., IoP, and decrements of peaks’ amplitudes, e.g., MSP. For each volunteer, we extract the eleven features on all repetitions, across all nine signals, including the two 3D signals (*x*, *y*, *z*) from the accelerometer and the gyroscope, total acceleration, exerted Force, and acc–gyro signal fusion.

### 2.4. Extracting Similarities

**Figure 5:**
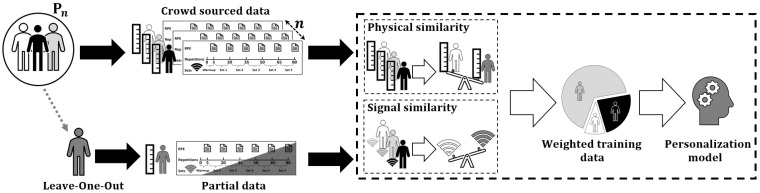
Visualization of the concept of personalizing general model using crowd-sourced wearables’ data.

To visualize the concept of our work, let us assume that a test subject is selected from a diverse population *P* of size *n*, as shown in Figure 5. Each member of the population reports their physical traits along with bicep concentration curl data signals. Meanwhile, the test subject provides only partial data, often one set of repetitions, of their bicep concentration curl data signals along with their physical traits. We measure the similarities between the test subject and members of the population so that the data from whom the test subject is similar gain more weight while training the model. We are keen to utilize two types of similarities, physical similarity and signal similarity, because previous studies have reported gains in performance when harnessing those similarities to weight data from the crowd [28,31].

We believe that combining both physical and signal similarities may further improve the personalized models’ performance. Therefore, in the second research question (RQ2), we measure and compare the performance between the personalized models trained using the weighted data and the cross-subject models. Furthermore, it comes to our minds that if we already possess and use a part of the test subject’s data to measure the similarities between the test subject and the crowd, then we may let the personalized models consume it in training to improve their learning. Therefore, in the third research question (RQ3), we also decided to let subject-specific models consume the same part of the test subject’s data; then, we compare the performances of personalized and subject-specific models. Moreover, we allow the subject-specific models to consume more of the test subject’s data if needed until it can reach the same performance of personalized models so that we quantify the amount of spared data by using personalized models.

#### 2.4.1. Measuring Physical Similarity

**Figure 6:**
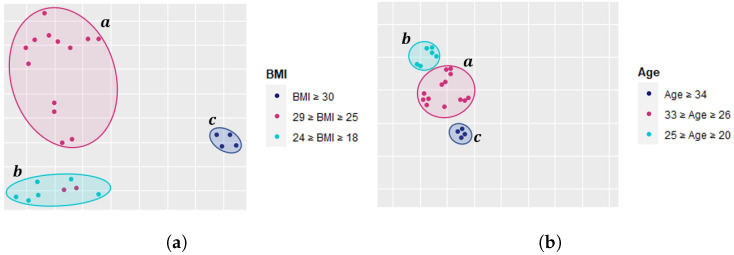
PCA plots showing signs of fatigue captured by the three fatigue-related features and BMI/age. (**a**) BMI perspective. (**b**) Age perspective.

Physical characteristics of people (e.g., age, weight, height, or BMI) vary from one person to another within a large population. Such differences can affect the way people move and perform physical activities. We believe that a user with different physical traits, e.g., age and BMI, may show signs of fatigue differently. At the same time, we expect groups of people who share similar physical traits to show similar signs of fatigue [80,81]. For example, let us capture the signs of fatigue using the three fatigue-related features, skewness, IoP, and MSP, and plot the principal component analysis (PCA). In Figure 6a, we often observe that individuals within specific limits of BMI values tend to share similar signs of fatigue. Moreover, Figure 6b shows similar observations where we use age instead of BMI. We can observe that individuals of certain ages tend to share similar signs of fatigue. This strengthens our hypothesis that if we construct the training data from individuals within the population who are more similar, it may reduce the inter-subject data variability and hence improve the performance of the fatigue detection model.

To compute the physical similarity value between a pair of users, we employ four types of physical traits: age, height, weight, and BMI. To limit the widespread of the values, due to subjects’ variations, we apply min–max normalization to each physical trait, on training data, to normalize each trait between 0 and 1. We combine these four traits per user to form a dedicated physical vector $V^{Phy}$ = {age, height, weight, BMI} representing their physical traits separately. We measure the distance $d^{Phy}$ between the physical traits $V^{Phy}$ of two users (q,p) based on the Manhattan distance, as shown in Equation (1). Previous works show that Manhattan distance is preferable to Euclidean for high dimensional data and if the dimensions are not comparable [82,83,84]. The physical similarity between users (q,p) is based on the universal law of generalization proposed in previous works [28,31,85,86], where distance and perceived similarity are related via an exponential function, as shown in Equation (2):

(1) $$d^{Phy}\left(q,p\right)=\sum_{k=1}^{4}\left|V_{q_{k}}^{Phy}-V_{p_{k}}^{Phy}\right|$$

(2) $$sim^{Phy}\left(q,p\right)=\frac{1}{e^{\gamma d^{Phy}\left(q,p\right)}}$$

where γ is an empirically determined scaling parameter that affects the shape of the exponential function. For example, $\lim_{\gamma \to \infty }sim^{Phy}\left(q,p\right)=0$, which indicates that as γ approaches infinity, the physical similarity approaches zero, causing more segregation between users. This can be a double-edged sword because as we segregate dissimilar users from each other, we may increase the segregation between similar users unintentionally. On the other hand, $\lim_{\gamma \to 0}sim^{Phy}\left(q,p\right)=1$, which indicates that as gamma approaches zero, the physical similarity approaches one, implying that all subjects show similar signs of fatigue; in other words, the changes in their data patterns are similar. Again, this is a double-edged scenario where we may unintentionally pull dissimilar users near to the similar users. Therefore, further investigation is required to estimate the optimal value of γ.

#### 2.4.2. Measuring Signal Similarity

In the context of signal similarities, we use one set of repetitions, approximately 20% of the subject’s data needed for the subject-specific models. We believe that users within the same population may show similar signs of fatigue, leading to similar changes in data patterns while performing the exercise. To compute the signal similarity value between a pair of users, we employ the 11 extracted features in Table 2 to form a dedicated signal vector $V^{Sig}$ = {min, max, ... , MSP} for each user. We measure the distance $d^{Sig}$ between the signal traits $V^{Sig}$ of two users (q,p) based on the Manhattan distance for all repetitions *l* = {1, 2, ... , L}, as shown in Equation (3).

(3) $$d^{Sig}\left(q,p\right)=\sum_{k=1}^{11}\sum_{l=1}^{L}\left|V_{q_{\left(k,l\right)}}^{Sig}-V_{p_{\left(k,l\right)}}^{Sig}\right|$$

(4) $$sim^{Sig}\left(q,p\right)=\frac{1}{e^{\gamma d^{Sig}\left(q,p\right)}}$$

The signal similarity between users (q,p) is based on the distance between their vectors, as shown in Equation (4).

#### 2.4.3. Measuring Total Similarity

We measure the total similarity $sim^{Total}$ between two users (q,p) by summing their weighted physical $sim^{Phy}\left(q,p\right)$ and signal $sim^{Sig}\left(q,p\right)$ similarities, as shown in Equation (5).

(5) $$sim^{Total}\left(q,p\right)=\alpha \times sim^{Phy}\left(q,p\right)+\beta \times sim^{Sig}\left(q,p\right)$$

where α+β=1. If α is greater than β, the physical similarity will contribute more than signal similarity in determining the total similarity value. On the other hand, if β is greater than α, the signal similarity will be the one that dominates the total similarity value. Therefore, in the first research question (RQ1), we further investigate the impact of (α,β) values on the performance of the personalized models. Moreover, we examine (γ) values to achieve the highest performance possible.

## 3. Experiments and Results

In this section, we describe the setup to evaluate the personalization approach in boosting the performance of cross-subject models. We start by listing the three research questions and the selected models for the experiment setup. Then, we identify the possible values for the parameters (α,β,γ) in Equations (2), (4) and (5).

- RQ1: What is the impact of the physical and signal parameters on the performance of the personalized biceps fatigue detection models?
- RQ2: Can the personalization approach improve the performance of cross-subject models in detecting biceps muscle fatigue?
- RQ3: Can the personalization approach reduce the consumption of the test subject’s data in comparison to subject-specific models?

We employ two models that utilize weighted data in the training to improve classification performance. Several works in the literature suggest using AdaBoost, a statistical boosting technique used as an ensemble method to reduce error in generalized models at the cost of weighing the training data [87,88,89]. Therefore, we use AdaBoost to take advantage of weighting the data, as mentioned in Section 2.4, while training these models. We select two models that have been previously used in HAR-related applications. The first model is the Decision Tree (DT); this model was used to count and classify ambulatory activities using eight plantar pressure sensors within smart shoes in a previous study [90]. The second model is the Artificial Neural Networks (ANN); this model was used to detect and count repetitions for complex physical exercises [91]. Specifically, we use Adaboost-backpropagation neural network, which is used in HAR-related works [92,93]. There are two reasons for choosing DT and ANN among several other machine learning algorithms. (1) The current work aims to mitigate the hindering effect of subject data variability on cross-subject models, which we previously encountered in our prior work [45]. Therefore, we used the same models as in our previous work to fairly examine whether our personalization approach can minimize such a problem. (2) We wanted to examine a previous study finding that AdaBoost often achieves higher improvement on weak classifiers such as DT than stronger ones such as ANN [92].

In this work, we construct the training dataset based on the similarity score between the test subject and the crowd. The data from whom the test subject has the highest similarity score are used in validation/ tuning the models, while the remaining data are used to train the models. On the other hand, the test dataset is unseen data collected from the test subject and used only to assess the performance of the models.

### 3.1. Examining the Hyper-Parameters in the Personalized Biceps Fatigue Detection Model

In this section, we state our motivation and approach for RQ1. Our motivation for RQ1 is that we believe the performance of users similarity-based models, such as those driven from the personalization approach, may degrade if the inadequate parameters are selected. In other words, valuable data from the crowd, e.g., similar users, may be discarded due to an unintended preference for the physical similarity over signal similarity and vice versa. Previous work shows that finding a balance between the extracted similarities is important to improve the accuracy of the models constantly [94].

Our approach to address RQ1 is that we examine the possible values for the parameters (α,β,γ) in Equations (2), (4) and (5) so that we observe the impact of these parameters on the models’ performance. We start with Equations (2) and (4) to examine γ, which has an arbitrary value between (0, *∞*). To observe the impact of the different γ values on the models’ performance, we employ each γ value to run two pairs of models: AdaBoost-DT and AdaBoost-ANN. The first pair is physical similarity-based models of AdaBoost-DT and AdaBoost-ANN, while the second pair is signal similarity-based models of AdaBoost-DT and AdaBoost-ANN. Then, we compute the changes in these models’ performance as the value of γ increases and select the gamma value that corresponds to the best performance. In Equation (5), we examine the physical α and signal β parameters while satisfying the condition α+β=1. We experiment with different α and β values and observe the effect by running two similarity-based models (AdaBoost-DT, AdaBoost-ANN). Then, we compute the models’ performance as the values of α and β change; then, we select the (α,β) values that correspond to the best performance.

#### 3.1.1. RQ1: What Is the Impact of the Physical and Signal Parameters on the Performance of the Personalized Biceps Fatigue Detection Models?

**Figure 7:**
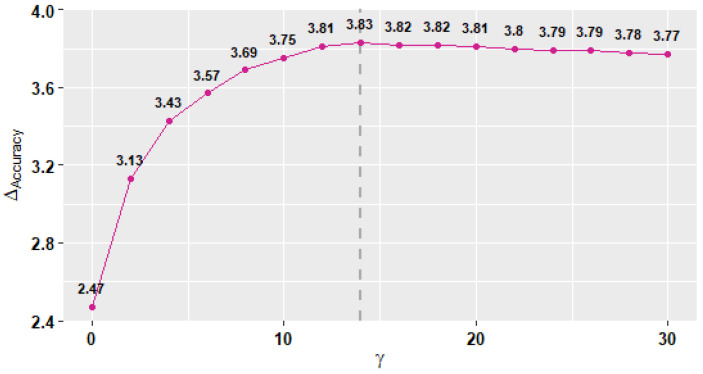
The average changes in both models’ accuracy as the value of γ increases.

Figure 7 shows the average changes in both models’ performance as the value of γ increases. The performance in this context is measured using accuracy. We use the accuracy at γ=0 as the reference point to measure the changes in accuracy (ΔAccuracy) as the γ value increases. We observe that ΔAccuracy increases as the γ value increases until both reach maximum values of 3.83 and 14 at the dashed line, respectively. Then, the changes in accuracy start to decline as the γ value continues to increase. Therefore, we select the γ=14 to let the models perform at maximum accuracy.

**Figure 8:**
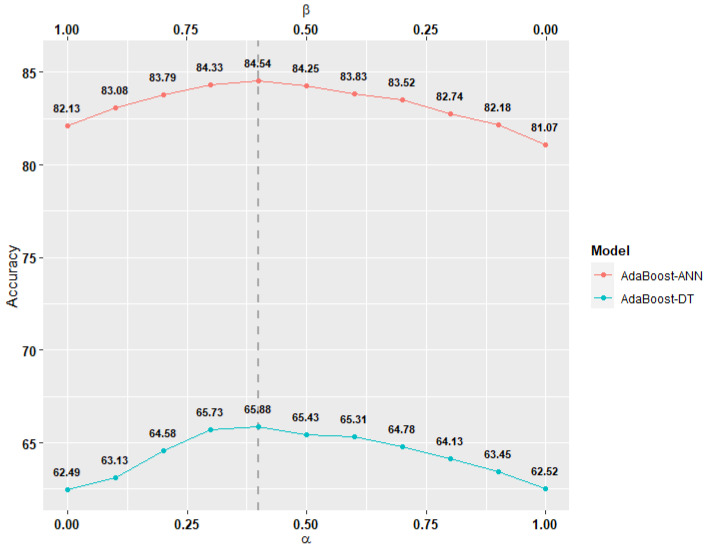
The average models’ accuracy as the values of α and β change.

Figure 8 shows the impact of the physical α and signal β parameters on models’ performance and there are three important findings in the figure. In the first finding, at α=0 and β=1, the models (AdaBoost-ANN, AdaBoost-DT) solely depend on the signal similarity, leading the training dataset, for these models, to be selected from the crowd with whom the test subject’s signal is similar while discarding the physical similarity. In this case, the two models (AdaBoost-ANN, AdaBoost-DT) achieve accuracy of 82.13% and 62.49%, respectively. In the second finding, at α=1 and β=0, the models (AdaBoost-ANN, AdaBoost-DT) solely depend on the physical similarity, leading the training dataset, for these models, to be selected from the crowd with whom the test subject’s physical traits is similar while discarding the signal similarity. In this case, the accuracy for the two models (AdaBoost-ANN, AdaBoost-DT) drops to 81.07% and 62.52%, respectively. In the third finding, at α=[0.25,0.50] and β=(1−α), the models (AdaBoost-ANN, AdaBoost-DT) depend on both physical and signal similarities; however, the training dataset, for these models, is selected from the crowd with whom the test subject’s is similar while prioritizing those with the highest signal similarity. In this case, we observe that the accuracy for the two models (AdaBoost-ANN, AdaBoost-DT) rises to reach 84.54% and 65.88%, respectively.

#### 3.1.2. RQ1 Conclusion

Our findings show that both physical and signal similarities are important. Moreover, the models’ performance reaches its peak when γ=14. The best selected values for α=0.4 and β=0.6 which we use for the rest of the evaluations.

### 3.2. Evaluating the Performance of Personalized Models

In this section, we state our motivation and approach for RQ2. Our motivation for RQ2 is that cross-subject models may seem preferable when it comes to a large number of users. However, a common trade-off for having cross-subject models is accuracy loss, especially for users with particular activity patterns. In other words, users who do not share enough similarities with the crowd may look as outliers where the cross-subject models are less accurate to detect their biceps muscle fatigue. We believe that adding weight to user’s data from whom the test subject is similar can improve model accuracy, including marginal users. Results of a previous study show that the personalization of cross-subject models constantly improves their accuracy compared with the standard cross-subject models [27].

(6) $$Accuracy=\frac{True(Fatigue+NonFatigue)}{True(Fatigue+NonFatigue)+False(Fatigue+NonFatigue)}$$

(7) $$Precision=\frac{True(Fatigue)}{True(Fatigue)+False(Fatigue)}$$

(8) $$Recall=\frac{True(Fatigue)}{True(Fatigue)+False(NonFatigue)}$$

(9) $$F1=\frac{2*Precision*Recall}{Precision+Recall}$$

Our approach to address RQ2 is that we use the 11 hand-crafted features along with the 2 similarity-based models (AdaBoost-DT, AdaBoost-ANN). We use these models to predict the Borg rating for each repetition, detecting whether a repetition contains fatigue or not. We run two experiments. In the first experiment, we set (α=0,β=0) in both models to mimic the standard cross-subject models. This means that the training dataset for these models is collected without considering any type of similarity between the crowd and the test subject. For this experiment, we use leave-one-out cross-validation (LOOCV), which is a K-fold cross-validation with K equal to the number of volunteers (K=25). In the second experiment, we set (α,β) to optimal values as identified in Section 3.1.1, leading the training dataset, for these models, to be selected based on physical and signal similarities, in addition to prioritizing data coming from users of highest signal similarity in the crowd. For each experiment, we calculate the accuracy using the confusion matrix shown in Table 3, where non-fatigue repetition represents a Borg score from 6 to 16, and fatigue status represents a Borg score from 17 to 20. We calculate the accuracy using Equation (6), precision using Equation (7), recall using Equation (8), and F1 using Equation (9).

#### 3.2.1. RQ2: Can the Personalization Approach Improve the Performance of Cross-Subject Models in Detecting Biceps Muscle Fatigue?

It is important to mention that we use one set of 15 repetitions approximately from the test subject’s data during the personalization of DT and ANN to measure the signal similarity between the test subject and individuals in the crowd. However, we do not include these 15 repetitions nor any data from the test subject in the training set. Our findings show that the personalization approach improves the accuracies for the models by 5.89% (DT) and by 3.38% (ANN), as shown in Table 4. The accuracy improved after prioritizing training data from the crowd based on the total similarity score, where individuals with high scores contribute more to the models’ training dataset. Moreover, we observe other improvements in terms of precision, recall, and F1-measure across the models. The results show that the personalization improves the DT model in terms of precision by (1.20%), recall by (4.51%), and F1-measure to (2.81%). On the other hand, the personalization improves the ANN model in terms of precision by (6.96%), recall by (4.55%), and F1-measure to (5.82%). Moreover, we observe that the standard cross-subject ANN model outperforms both DT models, which is expected in fatigue detection wherein ANN models often perform better than other cross-subject models [95].

#### 3.2.2. RQ2 Conclusion

Overall, our findings indicate that the personalization approach improves both models in terms of performance. For the DT model, the personalization improves its F1-measure from (60.94%) to (63.75%), while for the ANN model, the personalization improves its F1-measure from (75.82%) to (81.64%).

### 3.3. Examining the Consumption of the Test Subject’s Data in the Personalization Approach

In this section, we state our motivation and approach for RQ3. Our motivation for RQ3 is that subject-specific models are known for their high performance and demand of the test subject data. On the other hand, the cross-subject models are known for their relatively lower performance and no test subject data demand. We believe that the personalized approach can combine the best aspects of these two models. In other words, the personalization approach can improve the performance of the cross-subject models while consuming less test subject data than the subject-specific models. A previous work shows that adding a small amount of the test subject’s data to the training dataset for personalized models helps to improve the performance further closer to the subject-specific models [36].

Our approach to address RQ3 is that we utilized the 11 hand-crafted features and 4 models: subject-specific (DT, ANN) models and personalization (AdaBoost-DT, AdaBoost-ANN) models. We use these models to predict the Borg rating for each repetition to determine whether it is fatigue repetition or not. Similar to RQ2, we set (α,β) to optimal values so that the training dataset for these models is selected based on physical and signal similarities while prioritizing the signal similarity selection. Our experiment consists of seven runs where we incrementally add 10% of the test subject’s data to the training set after each run. This means, there is 0% of the test subject’s data added to the training dataset at the 1st run, while at the 7th run, there is 60% of the test subject’s data added to the training dataset. We stop at 60% of the test subject’s data to prevent overfitting the personalized model; otherwise, there will not be much of a difference between the subject-specific and the personalization approaches. Moreover, this allows us to measure the amount of the test subject’s data needed to improve the personalization models’ performance closer to the subject-specific models. Or, in other words, how little data we need from the test subject if we use the personalized models instead of subject-specific ones while keeping the accuracy relativity high. For each run, we calculate the accuracy using Equation (6) and accuracy gain ratio (AGR) using Equation (10).

(10) $$AGR=\frac{\Delta accuracy}{\mathrm{Test}\;\mathrm{subject}’s\;\mathrm{data}\;\left(\mathrm{repetitions}\right)}$$

#### 3.3.1. RQ3 Results: Can the Personalization Approach Reduce the Consumption of the Test Subject’s Data?

Our findings show that the more the test subject’s data are added to the training set, the higher the accuracy of the subject-specific and personalized models. Table 5 shows that the subject-specific DT model achieves an accuracy of 78.90% after consuming 40% of the test subject’s data. On the other hand, the personalization of the DT model achieves an accuracy of 76.08% after consuming 20% of the test subject’s data while compensating the rest of the training data from the similar users in the crowd. In other words, the subject-specific DT model requires twice the amount of test subject data, at 40%, to achieve similar accuracy to the personalized DT at 20% of the test subject’s data consumption with taking into consideration that the personalized DT model compensates the rest of the training data from the crowd. Moreover, our findings show that with 20% of the test subject’s data, the personalized DT model reaches the lowest accuracy gain ratio of 0.36% per test subject’s repetition while maintaining the highest accuracy gain of 5.44%.

Furthermore, the subject-specific ANN model achieves an accuracy of 92.99% after consuming 60% of the test subject’s data. On the other hand, the personalization of the ANN model achieves an accuracy of 92.74% after consuming 20% of the test subject’s data while compensating the rest of the training data from the similar users in the crowd. In other words, the subject-specific ANN model requires triple the amount of test subject’s data, at 60%, to achieve similar accuracy to the personalized ANN at 20% of the test subject’s data consumption with taking into consideration that the personalized ANN model compensates the rest of the training data from the crowd. Moreover, our findings show that with 20% of the test subject’s data, the personalized ANN model reaches the lowest accuracy gain ratio of ≈0.36% per test subject’s repetition while maintaining the highest accuracy gain of 5.37%.

#### 3.3.2. RQ3 Conclusion

Our findings show that the personalization approach may reduce the test subject’s data consumption by 33.3% up to 50.0% while reducing the accuracy gap compared to the subject-specific models.

## 4. Discussion

This section discusses our findings, points out work limitations, and proposes future works.

### 4.1. Findings Discussion

In RQ1, we observe that ΔAccuracy increases following a γ increase until it reaches a maximum of 3.83 at γ=14. Then, the accuracy starts to drop slightly with higher values of γ. Previous studies report similar behavior for the gamma parameter in their results sections [28,31]. They observe their models’ accuracy increases with an increase in γ value until γ reaches an optimal point, where their models then start losing accuracy. Although gamma’s behavior seems similar, the γ values are different and depend on the dataset. The reason behind gamma’s behavior resides in Equations (2) and (4) where we find that the physical and signal similarities approach zero as γ→∞. This means, if we keep increasing the gamma values, we will push the test subject further away from the crowd. In other words, we, unintentionally, decrease the possibility of finding similar users in the crowds, resulting in fewer similar data points, and hence smaller training data. On the other hand, when γ→0, the physical and signal similarities approach 1. This means, if we keep decreasing the gamma values, we will push the test subject closer toward the crowd, increasing the possibility of finding similar users. However, this can increase the risk of including low-quality data points from similar users with low ranks, which is usually the case in a cross-subject model; therefore, the accuracy often drops.

In RQ2, our findings show improvements in accuracy for both personalized models, AdaBoost-DT and AdaBoost-ANN, compared to the standard cross-subject ones, by 5.89% and 3.38%, respectively. Such improvements occur because the training datasets for the personalized models are selected from users who’s physical and signal traits are similar to the test subject. A previous study reports similar findings to ours, indicating that personalized models often perform +3% better than standard cross-subject models [27]. However, their proposed personalized model averages 0.78 for F1-score, while our AdaBoost-ANN model performs 3.64% better with an average of 81.64%±0.45 for F1-score. Overall, while this has been shown in previous works, our results help consolidate the benefits on relying on similarity as a method for boosting the performance of cross-subject models.

In RQ3, our finding indicates that the personalized models, AdaBoost-DT and AdaBoost-ANN, achieve comparable performance to subject-specific models while consuming 50.0% and 66.77% less test subject data. This is an important finding to motivate approaches that rely less on the data of the subject, particularly in cases where the test subject’s data are difficult to obtain or very limited. A previous study utilized a personalization approach to cut down the cost of data labeling by up to 90% for new users [37]. The study reports model accuracy between 77.7% and 83.4%. In contrast, we can observe that our personalized models, AdaBoost-DT and AdaBoost-ANN, achieve closer or higher accuracies at 76.08%±0.71 and 92.74%±0.49 at similar rates of 20% test data consumption, respectively, as shown in Table 6. This table shows the accuracy achieved by fatigue detection models including the cross-subject, subject-specific, and personalized models. As an implication, our findings suggest that personalized models are an effective approach to reduce data dependency—when data on the target subject is scarce—without severely compromising the model’s performance.

Moreover, we can observe that both of the personalization models achieve higher accuracies compared to the cross-subject models. However, we find that the personalization ANN models achieve lesser accuracy improvement than the personalization DT models. This agrees with previous work that shows that AdaBoost usually achieves higher improvement results on weak classifiers such as DT than stronger ones such as ANN [92].

### 4.2. Work Limitations

The first limitation of our work is the data size, which may affect the external validity of our study. While some HAR studies have opted to use public datasets, datasets with fatigue data are not common nor often available to the public [31,96]. Since we have to collect our fatigue data during the COVID-19 pandemic, it has been a daunting task due to social distancing and restrictive measures. Although our dataset may look small in size, we believe it is suitable for our research under such circumstances as other studies also collected their dataset with similar sizes to ours [97,98]. We agree that a bigger dataset is beneficial to our work, but we believe our experiments/approach can generate similar performance approximately.

The second limitation of our work is the reliance on the Apple Watch Series which uses the photoplethysmography (PPG) sensor to measure participants’ heart rate during the exercise. Although Apple Watch can provide the most accurate readings amongst the optical wrist wearables [99], previous works show that PPG often suffers from inaccuracies. This means our results may be indirectly impacted [100,101]; however, we believe such technology does not compromise our findings, especially in real-life applications. Previous works show that PPG achieves clinically acceptable accuracy and might be considered safe for rehabilitation training programs [102,103].

The third limitation of our work is the use of the Borg scale and the dumbbell weight. Although the Borg scale is often used in sports science, some studies are often cautious about its implications [104,105]. Using subjective measures such as the Borg scale to report RPE may introduce a dependency between the correctness of selected Borg rating and participants’ awareness. Therefore, we introduce the concept of the Borg scale to the participants in advance to avoid misevaluating their perceived exertion rate. On the other hand, dumbbell weight can directly vary the data points collected during the exercise because of each participant’s physical capacity or strength. A previous study shows lightweight dumbbells lead to a long recording session with many similar data points until participants reach fatigue [54]. In contrast, heavyweight dumbbells lead to shorter recording sessions with fewer data entries, which do not capture kinetic changes clearly throughout the exercise because participants reach fatigue quickly. Although we use a 4.5 kg weight dumbbell as recommended by previous studies, we believe having dumbbell weights will provide us with more information and different patterns of biceps muscle fatigue [51,52,53].

### 4.3. Future Works

The current work aims to mitigate the hindering effect of subject data variability from our prior works based on the Borg scale [45,55]. To achieve fairness in evaluation and continuity of our prior works, we use the Borg scale in our personalization approach. However, previous studies show the advantages of Electromyography (EMG) sensors in providing detailed information about muscle conditions during incremental exercises [97]. Moreover, a recent study that utilizes the EMG sensors shows an average accuracy of 97.8% in muscle fatigue detection during incremental exercises [106]. We believe utilizing such sensors can improve the performance of our personalization approach. However, collecting and processing EMG sensors data from bicep muscles will require effort, coding, and time; therefore, we opt to select EMG sensors in our future work.

Another future work that we are interested in is examining the personalization approach using deep learning such as Convolutional Neural Networks (CNNs) or Recurrent Neural Networks (RNN). A recent study shows a significant performance improvement (from 89.83% to 96.62%) after utilizing deep learning in HAR systems [107]. We opt to use DT and ANN models in the current study to examine whether our personalization approach can mitigate the hindering effect of subject data variability encountered in our previous works [45,55]. So, since our findings seem encouraging, we have decided to use CNN in our future work to examine our personalization approach further.

## 5. Conclusions

This work aims to mitigate the hindering effect of subject data variability in fatigue detection. We propose the personalization approach to utilize data from the crowd based on the total similarity score between the test subject and the crowd. Our dataset consists of 3750 concentration curl repetitions from 25 volunteers with ages and BMI ranging between 20–46 and 24–46, respectively. We compute the total similarity score between each individual in the crowd and the test subject based on the physical and signal similarity scores. Then, we extract a weighted dataset to train our models. Our findings show that the AdaBoost-DT model outperforms the cross-subject-DT model by 5.89%, while the AdaBoost-ANN model outperforms the cross-subject-ANN model by 3.38%. On the other hand, at 50.0% less of the test subject’s data consumption, our AdaBoost-DT model outperforms the subject-specific-DT model by 16%, while the AdaBoost-ANN model outperforms the subject-specific-ANN model by 10.33%. Our findings indicate that crowd data are usable to build personalized bicep fatigue detection models to prevent athletes from fatigue-induced injuries. Moreover, our personalization approach benefits real-life applications when the data from the test subject is unavailable or insufficient. We believe that our work is useful and represents a solid start for moving into real-world applications for detecting the fatigue level in bicep muscles using wearables’ data from the crowd.

## Figures and Tables

**Table 1:** Borg G.A. psychophysical bases of perceived exertion [49].

Perceived Exertion	Borg Rating	Examples
None	6	Reading a book, watching television
Very, very light	7 to 8	Tying shoes
Very light	9 to 10	Chores such as folding clothes that take little effort
Fairly light	11 to 12	Walking through a store (without speeding breath)
Somewhat hard	13 to 14	Brisk walking (mild effort and speeding breath)
Hard	15 to 16	Bicycling, swimming (effort and heart pounding)
Very hard	17 to 18	Intense activity but can be sustained
Very, very hard	19 to 20	Very intense activity that cannot be sustained

**Table 2:** Eleven hand-crafted features: eight HAR-related features and three fatigue-related features.

	Feature	Formula
Centralized	Minimum	$min=min_{i=1,...N}\left(x_{i}\right)$
	Maximum	$max=max_{i=1,...N}\left(x_{i}\right)$
	Mean	$\bar{x}=\frac{1}{N}\sum_{i=1}^{N}x_{i}$
	Median	$M=\left\{\begin{array}{c} x_{\frac{N+1}{2}},\;N\;\mathrm{odd}\\ \frac{1}{2}\left(x_{\frac{N}{2}}+x_{\frac{N}{2}+1}\right),N\;\mathrm{even} \end{array}\right.$
	Standard Deviation (SD)	$\sigma =\sqrt{\frac{1}{N}\sum_{i=1}^{N}{\left(x_{i}-\bar{x}\right)}^{2}}$
	Variance	$\sigma^{2}=\frac{1}{n}\sum_{i=1}^{n}{\left(x_{i}-\bar{x}\right)}^{2}$
	Kurtosis	$K=\frac{1}{N}\sum_{i=1}^{N}\frac{{\left(x_{i}-\bar{x}\right)}^{4}}{\sigma^{4}}$
	Root Mean Square (RMS)	$\mathrm{RMS}=\sqrt{\frac{1}{N}\sum_{i=1}^{N}{\left(x_{-}\bar{x}\right)}^{2}}$
Fatigue	Skewness	$\mathrm{Sk}=\frac{1}{N}\sum_{i=1}^{N}\frac{{\left(x_{i}-\bar{x}\right)}^{3}}{\sigma^{3}}$
	Interval of Peaks (IoP)	$IoP=T_{p}-T_{p-1}:p=2,...N$
	Mean Slope between Peaks (MSP)	$MSP=\frac{1}{N^{2}}\sum_{i=1}^{N}\sum_{j=1}^{N}\frac{p_{j}-p_{i}}{T_{p_{j}}-T_{p_{i}}}$

**Table 3:** Fatigue detection confusion matrix.

		Actual	
		Fatigue ∈ [17, 20]	Non-Fatigue ∈ [6, 16]
**Predict**	**Fatigue** ∈ **[17, 20]**	TRUE Fatigue	FALSE Fatigue
	**Non-Fatigue** ∈ **[6, 16]**	FALSE Non-Fatigue	TRUE Non-Fatigue

**Table 4:** Average precision, recall, and accuracy, with a CI of 95%, for detecting fatigue in biceps repetitions before and after the personalization of cross-subject models.

		Models					
		DT			ANN		
		Cross-Subject	Personalized	Δ	Cross-Subject	Personalized	Δ
**Precision**		60.57%±0.66	61.77%±0.58	1.20%	73.29%±0.43	80.25%±0.47	6.96%
**Recall**		61.32%±0.53	65.83%±0.49	4.51%	78.53%±0.39	83.08%±0.43	4.55%
**Accuracy**		60.08%±0.49	65.97%±0.67	5.89%	82.41%±0.58	85.79%±0.48	3.38%
**F1**		60.94%±0.59	63.75%±0.53	2.81%	75.82%±0.41	81.64%±0.45	5.82%

**Table 5:** The accuracy averages for the subject-specific and personalized models after adding 10% of the test subject’s data to the training set in each run incrementally. We include a version of this table with the confidence intervals in the Appendix A.

				Number of Biceps Repetitions Collected from the Test Subject (% of Used Test’s Data)						
				0	8	15	23	30	38	45
				(0%)	(10%)	(20%)	(30%)	(40%)	(50%)	(60%)
**Models**	**DT**	**Subject-specific**	Accuracy	15.34%	41.30%	58.40%	68.60%	78.90%	82.20%	84.03%
			Δaccuracy	-	25.96%	17.10%	10.20%	10.30%	3.30%	1.83%
			AGR	-	3.25%	1.14%	0.44%	0.34%	0.09%	0.04%
		**Personalized**	Accuracy	65.88%	70.64%	76.08%	77.77%	79.15%	79.55%	79.91%
			Δaccuracy	-	4.76%	5.44%	1.69%	1.38%	0.40%	0.36%
			AGR	-	0.60%	0.36%	0.07%	0.05%	0.01%	0.01%
	**ANN**	**Subject-specific**	Accuracy	55.23%	62.48%	74.68%	82.95%	87.37%	90.45%	92.99%
			Δaccuracy	-	7.25%	12.20%	8.27%	4.42%	3.08%	2.54%
			AGR	-	0.91%	0.81%	0.36%	0.15%	0.08%	0.06%
		**Personalized**	Accuracy	84.54%	87.37%	92.74%	93.56%	93.88%	94.25%	94.78%
			Δaccuracy	-	2.83%	5.37%	0.82%	0.32%	0.37%	0.53%
			AGR	-	0.35%	0.36%	0.04%	0.01%	0.01%	0.01%

**Table 6:** Percent accuracy achieved on, with a CI of 95%, the cross-subject, subject-specific, and personalization models.

(% of Used Test’s Data)			Accuracy	Δ Accuracy		
				Cross-Subject	Personalized	Subject-Specific
**Models**	**DT**	Cross-Subject (0%)	60.08%±0.49	-	−16.00%	−28.67%
		Personalized (20%)	76.08%±0.71	16.00%	-	−12.67%
		Subject-Specific (100%)	88.75%±0.59	28.67%	12.67%	-
	**ANN**	Cross-Subject (0%)	82.41%±0.58	-	−10.33%	−16.89%
		Personalized (20%)	92.74%±0.49	10.33%	-	−6.56%
		Subject-Specific (100%)	99.30%±0.37	16.89%	6.56%	-

## Data Availability

The data used in the study is made publicly available at—https://zenodo.org/record/3698242#.XmFZ5qhKguU, accessed on 20 December 2021.

## Appendix A

**Table A1:** The accuracy averages for the subject-specific and personalized models after adding 10% of the test subject’s data to the training set in each run incrementally.

				Number of Biceps Repetitions Collected from the Test Subject (% of Used Test’s Data)						
				0	8	15	23	30	38	45
				(0%)	(10%)	(20%)	(30%)	(40%)	(50%)	(60%)
**Models**	**DT**	**Subject-specific**	Accuracy	15.34%±0.83	41.30%±0.78	58.40%±0.71	68.60%±0.63	78.90%±0.57	82.20%±0.48	84.03%±0.41
			Δaccuracy	-	25.96%	17.10%	10.20%	10.30%	3.30%	1.83%
			AGR	-	3.25%	1.14%	0.44%	0.34%	0.09%	0.04%
		**Personalized**	Accuracy	65.88%±0.65	70.64%±0.68	76.08%±0.71	77.77%±0.66	79.15%±0.59	79.55%±0.51	79.91%±0.47
			Δaccuracy	-	4.76%	5.44%	1.69%	1.38%	0.40%	0.36%
			AGR	-	0.60%	0.36%	0.07%	0.05%	0.01%	0.01%
	**ANN**	**Subject-specific**	Accuracy	55.23%±0.79	62.48%±0.72	74.68%±0.64	82.95%±0.58	87.37%±0.45	90.45%±0.47	92.99%
			Δaccuracy	-	7.25%	12.20%	8.27%	4.42%	3.08%	2.54%
			AGR	-	0.91%	0.81%	0.36%	0.15%	0.08%	0.06%±0.33
		**Personalized**	Accuracy	84.54%±0.63	87.37%±0.55	92.74%±0.49	93.56%±0.42	93.88%±0.48	94.25%±0.45	94.78%±0.39
			Δaccuracy	-	2.83%	5.37%	0.82%	0.32%	0.37%	0.53%
			AGR	-	0.35%	0.36%	0.04%	0.01%	0.01%	0.01%

## References

[1] Enoka R.M., Duchateau J. (2008). Muscle fatigue: What, why and how it influences muscle function. J. Physiol..

[2] Opar D., Williams M., Shield A. (2012). Hamstring Strain Injuries Factors that Lead to Injury and Re-Injury. Sport. Med..

[3] Mueller-Wohlfahrt H.W., Haensel L., Mithoefer K., Ekstrand J., English B., McNally S., Orchard J., van Dijk C.N., Kerkhoffs G.M., Schamasch P. (2013). Terminology and classification of muscle injuries in sport: The Munich consensus statement. Br. J. Sport. Med..

[4] Kellmann M. (2010). Preventing overtraining in athletes in high-intensity sports and stress/recovery monitoring. Scand. J. Med. Sci. Sports.

[5] Thalman C.M., Lam Q.P., Nguyen P.H., Sridar S., Polygerinos P. A Novel Soft Elbow Exosuit to Supplement Bicep Lifting Capacity. Proceedings of the 2018 IEEE/RSJ International Conference on Intelligent Robots and Systems (IROS).

[6] Stoudemire N.M., Wideman L., Pass K.A., Mcginnes C.L., Gaesser G.A., Weltman A. (1996). The validity of regulating blood lactate concentration during running by ratings of perceived exertion. Med. Sci. Sports Exerc..

[7] Crewe H., Tucker R., Noakes T.D. (2008). The rate of increase in rating of perceived exertion predicts the duration of exercise to fatigue at a fixed power output in different environmental conditions. Eur. J. Appl. Physiol..

[8] Bosquet L., Léger L., Legros P. (2001). Blood lactate response to overtraining in male endurance athletes. Eur. J. Appl. Physiol..

[9] Kobayashi Y., Takeuchi T., Hosoi T., Yoshizaki H., Loeppky J.A. (2005). Effect of a marathon run on serum lipoproteins, creatine kinase, and lactate dehydrogenase in recreational runners. Res. Q. Exerc. Sport.

[10] Billat L.V., Koralsztein J.P. (1996). Significance of the velocity at VO 2max and time to exhaustion at this velocity. Sports Med..

[11] Cannon D.T., White A.C., Andriano M.F., Kolkhorst F.W., Rossiter H.B. (2011). Skeletal muscle fatigue precedes the slow component of oxygen uptake kinetics during exercise in humans. J. Physiol..

[12] Robson-Ansley P.J., Gleeson M., Ansley L. (2009). Fatigue management in the preparation of Olympic athletes. J. Sports Sci..

[13] Lee S.M., Yoon S.M., Cho H. Human activity recognition from accelerometer data using Convolutional Neural Network. Proceedings of the 2017 IEEE International Conference on Big Data and Smart Computing (BigComp).

[14] Nithya N., Nallavan G. Role of Wearables in Sports based on Activity recognition and biometric parameters: A Survey. Proceedings of the 2021 International Conference on Artificial Intelligence and Smart Systems (ICAIS).

[15] Op De Beéck T., Meert W., Schütte K., Vanwanseele B., Davis J. Fatigue prediction in outdoor runners via machine learning and sensor fusion. Proceedings of the 24th ACM SIGKDD International Conference on Knowledge Discovery & Data Mining.

[16] Barshan B., Yüksek M.C. (2014). Recognizing daily and sports activities in two open source machine learning environments using body-worn sensor units. Comput. J..

[17] Shoaib M., Bosch S., Incel O.D., Scholten H., Havinga P.J. (2014). Fusion of smartphone motion sensors for physical activity recognition. Sensors.

[18] Lockhart J.W., Weiss G.M. Limitations with activity recognition methodology & data sets. Proceedings of the 2014 ACM International Joint Conference on Pervasive and Ubiquitous Computing: Adjunct Publication.

[19] Barshan B., Yurtman A. (2016). Investigating inter-subject and inter-activity variations in activity recognition using wearable motion sensors. Comput. J..

[20] Kristiansen M., Madeleine P., Hansen E.A., Samani A. (2015). Inter-subject variability of muscle synergies during bench press in power lifters and untrained individuals. Scand. J. Med. Sci. Sports.

[21] Mourão-Miranda J., Hardoon D.R., Hahn T., Marquand A.F., Williams S.C., Shawe-Taylor J., Brammer M. (2011). Patient classification as an outlier detection problem: An application of the one-class support vector machine. Neuroimage.

[22] Kobsar D., Ferber R. (2018). Wearable sensor data to track subject-specific movement patterns related to clinical outcomes using a machine learning approach. Sensors.

[23] Lubetzky-Vilnai A., Ciol M., McCoy S.W. (2014). Statistical analysis of clinical prediction rules for rehabilitation interventions: Current state of the literature. Arch. Phys. Med. Rehabil..

[24] Fredriksson T., Mattos D.I., Bosch J., Olsson H.H. (2020). Data labeling: An empirical investigation into industrial challenges and mitigation strategies. Proceedings of the International Conference on Product-Focused Software Process Improvement.

[25] Nweke H.F., Teh Y.W., Al-Garadi M.A., Alo U.R. (2018). Deep learning algorithms for human activity recognition using mobile and wearable sensor networks: State of the art and research challenges. Expert Syst. Appl..

[26] Fallahzadeh R., Ghasemzadeh H. Personalization without user interruption: Boosting activity recognition in new subjects using unlabeled data. Proceedings of the 8th International Conference on Cyber-Physical Systems.

[27] Sztyler T., Stuckenschmidt H. Online personalization of cross-subjects based activity recognition models on wearable devices. Proceedings of the 2017 IEEE International Conference on Pervasive Computing and Communications (PerCom).

[28] Lane N.D., Xu Y., Lu H., Hu S., Choudhury T., Campbell A.T., Zhao F. Enabling large-scale human activity inference on smartphones using community similarity networks (csn). Proceedings of the 13th International Conference on Ubiquitous Computing.

[29] Chen Y., Wang J., Huang M., Yu H. (2019). Cross-position activity recognition with stratified transfer learning. Pervasive Mob. Comput..

[30] Khan M.A.A.H., Roy N., Misra A. Scaling human activity recognition via deep learning-based domain adaptation. Proceedings of the 2018 IEEE international conference on pervasive computing and communications (PerCom).

[31] Ferrari A., Micucci D., Mobilio M., Napoletano P. (2020). On the personalization of classification models for human activity recognition. IEEE Access.

[32] Palmius N., Saunders K.E., Carr O., Geddes J.R., Goodwin G.M., De Vos M. (2018). Group-personalized regression models for predicting mental health scores from objective mobile phone data streams: Observational study. J. Med Internet Res..

[33] Huang Z., Niu Q., You I., Pau G. (2021). Acceleration Feature Extraction of Human Body Based on Wearable Devices. Energies.

[34] Janidarmian M., Roshan Fekr A., Radecka K., Zilic Z. (2017). A comprehensive analysis on wearable acceleration sensors in human activity recognition. Sensors.

[35] Mannini A., Intille S.S. (2018). Classifier personalization for activity recognition using wrist accelerometers. IEEE J. Biomed. Health Informatics.

[36] Weiss G.M., Lockhart J. The impact of personalization on smartphone-based activity recognition. Proceedings of the Workshops at the Twenty-Sixth AAAI Conference on Artificial Intelligence.

[37] Hong J.H., Ramos J., Dey A.K. (2016). Toward Personalized Activity Recognition Systems With a Semipopulation Approach. IEEE Trans. Hum. -Mach. Syst..

[38] Igual R., Medrano C., Plaza I. (2015). A comparison of public datasets for acceleration-based fall detection. Med. Eng. Phys..

[39] Steffen L.M., Arnett D.K., Blackburn H., Shah G., Armstrong C., Luepker R.V., Jacobs J.D. (2006). Population trends in leisure-time physical activity: Minnesota Heart Survey, 1980–2000. Med. Sci. Sports Exerc..

[40] Troiano R.P., Berrigan D., Dodd K.W., Masse L.C., Tilert T., McDowell M. (2008). Physical activity in the United States measured by accelerometer. Med. Sci. Sports Exerc..

[41] Mair S.D., Seaber A.V., Glisson R.R., Garrett W.E. (1996). The role of fatigue in susceptibility to acute muscle strain injury. Am. J. Sports Med..

[42] Garrett W.E. (1996). Muscle strain injuries. Am. J. Sports Med..

[43] Nesterenko S., Domire Z.J., Morrey B.F., Sanchez-Sotelo J. (2010). Elbow strength and endurance in patients with a ruptured distal biceps tendon. J. Shoulder Elb. Surg..

[44] Hopkins W.G., Marshall S.W., Quarrie K.L., Hume P.A. (2007). Risk factors and risk statistics for sports injuries. Clin. J. Sport Med..

[45] Elshafei M., Shihab E. (2021). Towards Detecting Biceps Muscle Fatigue in Gym Activity Using Wearables. Sensors.

[46] Apriantono T., Nunome H., Ikegami Y., Sano S. (2006). The effect of muscle fatigue on instep kicking kinetics and kinematics in association football. J. Sports Sci..

[47] Sanchez-Medina L., González-Badillo J.J. (2011). Velocity loss as an indicator of neuromuscular fatigue during resistance training. Med. Sci. Sports Exerc..

[48] Coelho A.C., Cannon D.T., Cao R., Porszasz J., Casaburi R., Knorst M.M., Rossiter H.B. (2015). Instantaneous quantification of skeletal muscle activation, power production, and fatigue during cycle ergometry. J. Appl. Physiol..

[49] Borg G.A. (1982). Psychophysical bases of perceived exertion. Med. Sci. Sports Exerc..

[50] Yoo S., Ackad C., Heywood T., Kay J. Evaluating the actual and perceived exertion provided by virtual reality games. Proceedings of the 2017 CHI Conference Extended Abstracts on Human Factors in Computing Systems.

[51] Liao F., Zhang X., Cao C., Hung I.Y.J., Chen Y., Jan Y.K. (2021). Effects of muscle fatigue and recovery on complexity of surface electromyography of Biceps Brachii. Entropy.

[52] Hwang H.J., Chung W.H., Song J.H., Lim J.K., Kim H.S. (2016). Prediction of biceps muscle fatigue and force using electromyography signal analysis for repeated isokinetic dumbbell curl exercise. J. Mech. Sci. Technol..

[53] Bergquist R., Iversen V.M., Mork P.J., Fimland M.S. (2018). Muscle activity in upper-body single-joint resistance exercises with elastic resistance bands vs. free weights. J. Hum. Kinet..

[54] Reis V.M., Garrido N.D., Vianna J., Sousa A.C., Alves J.V., Marques M.C. (2017). Energy cost of isolated resistance exercises across low-to high-intensities. PLoS ONE.

[55] Elshafei M., Costa D.E., Shihab E. (2021). On the Impact of Biceps Muscle Fatigue in Human Activity Recognition. Sensors.

[56] Borg G. (1998). Borg’s Perceived Exertion and Pain Scales..

[57] Adirim T.A., Cheng T.L. (2003). Overview of injuries in the young athlete. Sports Med..

[58] Burt C.W., Overpeck M.D. (2001). Emergency visits for sports-related injuries. Ann. Emerg. Med..

[59] Green B., Pizzari T. (2017). Calf muscle strain injuries in sport: A systematic review of risk factors for injury. Br. J. Sports Med..

[60] Prentice A.M., Jebb S.A. (2001). Beyond body mass index. Obes. Rev..

[61] Borga M., West J., Bell J.D., Harvey N.C., Romu T., Heymsfield S.B., Leinhard O.D. (2018). Advanced body composition assessment: From body mass index to body composition profiling. J. Investig. Med..

[62] Burkhauser R.V., Cawley J. (2008). Beyond BMI: The value of more accurate measures of fatness and obesity in social science research. J. Health Econ..

[63] Jones B.H., Hauret K.G., Dye S.K., Hauschild V.D., Rossi S.P., Richardson M.D., Friedl K.E. (2017). Impact of physical fitness and body composition on injury risk among active young adults: A study of army trainees. J. Sci. Med. Sport.

[64] Janssen I., Katzmarzyk P.T., Ross R. (2002). Body mass index, waist circumference, and health risk: Evidence in support of current National Institutes of Health guidelines. Arch. Intern. Med..

[65] Whittaker R.L., Sonne M.W., Potvin J.R. (2019). Ratings of perceived fatigue predict fatigue induced declines in muscle strength during tasks with different distributions of effort and recovery. J. Electromyogr. Kinesiol..

[66] Alarfaj M., Qian Y., Liu H. Detection of Human Body Movement Patterns Using IMU and Barometer. Proceedings of the 2020 International Conference on Communications, Signal Processing, and their Applications (ICCSPA).

[67] Webber M., Rojas R.F. (2021). Human Activity Recognition with Accelerometer and Gyroscope: A Data Fusion Approach. IEEE Sens. J..

[68] Li Q., Li R., Ji K., Dai W. Kalman filter and its application. Proceedings of the 2015 8th International Conference on Intelligent Networks and Intelligent Systems (ICINIS).

[69] Wichit N., Choksuriwong A. (2015). Multi-sensor data fusion model based Kalman filter using fuzzy logic for human activity detection. Int. J. Inf. Electron. Eng..

[70] Palumbo F., Gallicchio C., Pucci R., Micheli A. (2016). Human activity recognition using multisensor data fusion based on reservoir computing. J. Ambient. Intell. Smart Environ..

[71] Wang Y., Cang S., Yu H. (2018). A data fusion-based hybrid sensory system for older people’s daily activity and daily routine recognition. IEEE Sens. J..

[72] Nweke H.F., Teh Y.W., Mujtaba G., Al-Garadi M.A. (2019). Data fusion and multiple classifier systems for human activity detection and health monitoring: Review and open research directions. Inf. Fusion.

[73] Ferrari A., Micucci D., Mobilio M., Napoletano P. Hand-crafted features vs residual networks for human activities recognition using accelerometer. Proceedings of the 2019 IEEE 23rd International Symposium on Consumer Technologies (ISCT).

[74] Vanrell S.R., Milone D.H., Rufiner H.L. (2017). Assessment of homomorphic analysis for human activity recognition from acceleration signals. IEEE J. Biomed. Health Inform..

[75] Bianco S., Napoletano P., Schettini R. Multimodal car driver stress recognition. Proceedings of the 13th EAI International Conference on Pervasive Computing Technologies for Healthcare.

[76] Ji Q., Lan P., Looney C. (2006). A probabilistic framework for modeling and real-time monitoring human fatigue. IEEE Trans. Syst. Man Cybern.-Part A Syst. Hum..

[77] Mallis M.M., Mejdal S., Nguyen T.T., Dinges D.F. (2004). Summary of the key features of seven biomathematical models of human fatigue and performance. Aviat. Space Environ. Med..

[78] Sant’Ana M., Li G., Zhang H. A decentralized sensor fusion approach to human fatigue monitoring in maritime operations. Proceedings of the 2019 IEEE 15th International Conference on Control and Automation (ICCA).

[79] Aghamohammadi-Sereshki A., Bayazi M.J.D., Ghomsheh F.T., Amirabdollahian F. Investigation of Fatigue Using Different EMG Features. Proceedings of the 2019 IEEE 16th International Conference on Rehabilitation Robotics (ICORR).

[80] Morgan P.T., Smeuninx B., Breen L. (2020). Exploring the impact of obesity on skeletal muscle function in older age. Front. Nutr..

[81] Tomlinson D.J., Erskine R.M., Morse C.I., Pappachan J.M., Sanderson-Gillard E., Onambélé-Pearson G.L. (2021). The combined effects of obesity and ageing on skeletal muscle function and tendon properties in vivo in men. Endocrine.

[82] Malkauthekar M. Analysis of Euclidean distance and Manhattan distance measure in Face recognition. Proceedings of the Third International Conference on Computational Intelligence and Information Technology (CIIT 2013).

[83] Aggarwal C.C., Hinneburg A., Keim D.A. On the surprising behavior of distance metrics in high dimensional space. Proceedings of the International Conference on Database Theory.

[84] Shirkhorshidi A.S., Aghabozorgi S., Wah T.Y. (2015). A comparison study on similarity and dissimilarity measures in clustering continuous data. PLoS ONE.

[85] Shepard R.N. (1987). Toward a universal law of generalization for psychological science. Science.

[86] Tenenbaum J.B., Griffiths T.L. (2001). Generalization, similarity, and Bayesian inference. Behav. Brain Sci..

[87] Walse K.H., Dharaskar R.V., Thakare V.M. (2017). A study on the effect of adaptive boosting on performance of classifiers for human activity recognition. Proceedings of the International Conference on Data Engineering and Communication Technology.

[88] Vezhnevets A., Vezhnevets V. Modest AdaBoost-teaching AdaBoost to generalize better. Proceedings of the Graphicon.

[89] Solomatine D.P., Shrestha D.L. AdaBoost. RT: A boosting algorithm for regression problems. Proceedings of the 2004 IEEE International Joint Conference on Neural Networks (IEEE Cat. No. 04CH37541).

[90] Jeong G.M., Truong P.H., Choi S.I. (2017). Classification of three types of walking activities regarding stairs using plantar pressure sensors. IEEE Sens. J..

[91] Soro A., Brunner G., Tanner S., Wattenhofer R. (2019). Recognition and repetition counting for complex physical exercises with deep learning. Sensors.

[92] Subasi A., Dammas D.H., Alghamdi R.D., Makawi R.A., Albiety E.A., Brahimi T., Sarirete A. (2018). Sensor based human activity recognition using adaboost ensemble classifier. Procedia Comput. Sci..

[93] Fang H., He L., Si H., Liu P., Xie X. (2014). Human activity recognition based on feature selection in smart home using back-propagation algorithm. ISA Trans..

[94] Zhu Y., Wang C., Zhang J., Xu J. Human activity recognition based on similarity. Proceedings of the 2014 IEEE 17th International Conference on Computational Science and Engineering.

[95] Ghazal M., Haeyeh Y.A., Abed A., Ghazal S. Embedded Fatigue Detection Using Convolutional Neural Networks with Mobile Integration. Proceedings of the 2018 6th International Conference on Future Internet of Things and Cloud Workshops (FiCloudW).

[96] Lin C.Y., Marculescu R. Model personalization for human activity recognition. Proceedings of the 2020 IEEE International Conference on Pervasive Computing and Communications Workshops (PerCom Workshops).

[97] Jebelli H., Lee S., Mutis I., Hartmann T. (2019). Feasibility of Wearable Electromyography (EMG) to Assess Construction Workers’ Muscle Fatigue. Proceedings of the Advances in Informatics and Computing in Civil and Construction Engineering.

[98] Wan S., Qi L., Xu X., Tong C., Gu Z. (2020). Deep learning models for real-time human activity recognition with smartphones. Mob. Netw. Appl..

[99] Gillinov S., Etiwy M., Wang R., Blackburn G., Phelan D., Gillinov A.M., Houghtaling P., Javadikasgari H., Desai M.Y. (2017). Variable accuracy of wearable heart rate monitors during aerobic exercise. Med. Sci. Sports Exerc..

[100] Gil E., Orini M., Bailon R., Vergara J.M., Mainardi L., Laguna P. (2010). Photoplethysmography pulse rate variability as a surrogate measurement of heart rate variability during non-stationary conditions. Physiol. Meas..

[101] Schäfer A., Vagedes J. (2013). How accurate is pulse rate variability as an estimate of heart rate variability?: A review on studies comparing photoplethysmographic technology with an electrocardiogram. Int. J. Cardiol..

[102] Shcherbina A., Mattsson C.M., Waggott D., Salisbury H., Christle J.W., Hastie T., Wheeler M.T., Ashley E.A. (2017). Accuracy in wrist-worn, sensor-based measurements of heart rate and energy expenditure in a diverse cohort. J. Pers. Med..

[103] Falter M., Budts W., Goetschalckx K., Cornelissen V., Buys R. (2019). Accuracy of Apple Watch Measurements for heart rate and energy expenditure in patients with cardiovascular disease: Cross-Sectional Study. JMIR mHealth uHealth.

[104] Arney B.E., Glover R., Fusco A., Cortis C., de Koning J.J., van Erp T., Jaime S., Mikat R.P., Porcari J.P., Foster C. (2019). Comparison of RPE (rating of perceived exertion) scales for session RPE. Int. J. Sport. Physiol. Perform..

[105] Sala E., Lopomo N.F., Tomasi C., Romagnoli F., Morotti A., Apostoli P., De Palma G. (2021). Importance of Work-Related Psychosocial Factors in Exertion Perception Using the Borg Scale Among Workers Subjected to Heavy Physical Work. Front. Public Health.

[106] Wang S., Tang H., Wang B., Mo J. (2021). A Novel Approach to Detecting Muscle Fatigue Based on sEMG by Using Neural Architecture Search Framework. IEEE Trans. Neural Netw. Learn. Syst..

[107] Gil-Martín M., San-Segundo R., Fernandez-Martinez F., Ferreiros-López J. (2020). Improving physical activity recognition using a new deep learning architecture and post-processing techniques. Eng. Appl. Artif. Intell..

